# Novel nanostructure approach for antibiotic decomposition in a spinning disc photocatalytic reactor

**DOI:** 10.1038/s41598-024-61340-8

**Published:** 2024-05-08

**Authors:** Saeid Fallahizadeh, Mahmood Reza Rahimi, Mitra Gholami, Ali Esrafili, Mahdi Farzadkia, Majid Kermani

**Affiliations:** 1 Research Center for Environmental Health Technology, Iran University of Medical Sciences, Tehran, Iran; 2 Department of Environmental Health Engineering, School of Public Health, Iran University of Medical Sciences, Tehran, Iran; 3 Process Intensification Laboratory, Department of Chemical Engineering, Yasouj University, Yasouj, Iran

**Keywords:** Amoxicillin, Core–shell, Photodecomposition, SDPR, Thin film, Environmental sciences, Chemistry

## Abstract

Conventional wastewater treatment processes are often unable to remove antibiotics with resistant compounds and low biological degradation. The need for advanced and sustainable technologies to remove antibiotics from water sources seems essential. In this regard, the effectiveness of a spinning disc photocatalytic reactor (SDPR) equipped with a visible light-activated Fe_3_O_4_@SiO_2_-NH_2_@CuO/ZnO core–shell (FSNCZ CS) thin film photocatalyst was investigated for the decomposition of amoxicillin (AMX), a representative antibiotic. Various characterization techniques, such as TEM, FESEM, EDX, AFM, XRD, and UV–Vis-DRS, were employed to study the surface morphology, optoelectronic properties, and nanostructure of the FSNCZ CS. Key operating parameters such as irradiation time, pH, initial AMX concentration, rotational speed, and solution flow rate were fine-tuned for optimization. The results indicated that the highest AMX decomposition (98.7%) was attained under optimal conditions of 60 min of irradiation time, a rotational speed of 350 rpm, a solution flow rate of 0.9 L/min, pH of 5, and an initial AMX concentration of 20 mg/L. Moreover, during the 60 min irradiation time, more than 69.95% of chemical oxygen demand and 61.2% of total organic carbon were removed. After the photocatalytic decomposition of AMX, there is a substantial increase in the average oxidation state and carbon oxidation state in SDPR from 1.33 to 1.94 and 3.2, respectively. Active species tests confirmed that ·OH and ·O_2_^−^ played a dominant role in AMX decomposition. The developed SDPR, which incorporates a reusable and robust FSNCZ CS photocatalyst, demonstrates promising potential for the decomposition of organic compounds.

## Introduction

Water is a vital resource for maintaining the health and survival of all living organisms on Earth. With the increasing global population and industrial development, the need for clean and usable water has grown. However, unfortunately, due to human activities and undesirable practices, our waters are becoming increasingly polluted^1,2^. One of the common pollutants in aquatic environments is antibiotics^3^. Exposure to antibiotics in the environment, particularly in drinking water and food crops, has the potential to inflict severe harm on public health, leading to fatal consequences^4^. Antibiotics are chemical compounds used to destroy and reduce the growth of bacteria^5–7^. Amoxicillin is an antibiotic from the penicillin family and β-lactam class that is widely used to treat bacterial infections^8^. The environmental effects of amoxicillin as a chemical drug depend on various environmental factors. The environmental effects of amoxicillin include contamination of water sources, drug resistance, changes in microbial population composition, impact on beneficial microorganisms, and toxicity^9^. Because of its physical and chemical properties, amoxicillin can easily enter water sources and cause pollution in water bodies and wastewater outlets.

Several technologies have been developed to address the removal of antibiotics from water, including biological methods and advanced oxidation processes (AOPs)^10,11^. Due to their simplicity and high oxidizing potential, advanced oxidation processes have recently garnered significant attention^12^. Among these, photocatalysis, particularly with solar light-activated catalysts, has garnered considerable attention because of its cost-effectiveness, high efficiency, and environmentally friendly nature^13,14^. The configuration of photocatalytic reactors can vary depending on the type of catalyst used. Here are some common configurations based on different catalyst types, including slurry photocatalytic reactor and immobilized photocatalytic reactor^15^. In the slurry photocatalytic reactor setup, the catalyst substance is dispersed in a fluid solution. The reactor typically consists of a vessel in which the catalyst particles are dispersed and mixed with the reactant solution. It may involve stirring or recirculation to ensure proper mixing and contact between the catalyst and the reactants. One drawback of slurry reactors is the need for separation and recovery of the suspended catalyst particles after the reaction, which can be challenging and may lead to catalyst loss^16^. Additionally, the presence of solid particles in the reactor can cause issues such as fouling or clogging of equipment, requiring frequent maintenance and cleaning^16,17^. In the immobilized photocatalytic reactor configuration, the catalyst material is immobilized onto a solid support, such as a membrane, glass beads, or fibers. The reactants flow over or through the immobilized catalyst, allowing efficient interaction and photocatalytic decomposition^18^. One drawback of immobilized reactors is the potential mass transfer restrictions due to the reduced surface area or limited exposure of the reactants to the catalyst^19^. Catalyst fouling or deactivation can also occur over time, necessitating periodic cleaning or catalyst replacement. Immobilization may also reduce the accessibility of the catalyst to light, thereby affecting the overall photocatalytic activity^20–22^.

A spinning disc photocatalytic reactor offers some advantages over slurry and immobilized catalyst systems. It overcomes the drawbacks associated with catalyst loss in slurry reactors by immobilizing the photocatalyst on a spinning disc, minimizing the risk of catalyst particles being carried away with the treated water^23^. In addition, the spinning disc reactor provides enhanced mass transfer by improving the dispersion of reactants and maximizing contact between the catalyst surface and the solution. This can lead to higher reaction rates and improved efficiency compared with slurry or immobilized catalyst systems^24^.

Heterogeneous photocatalysis is a promising alternative for eliminating drug residues from water bodies because of its high oxidation power, efficient antibiotic removal, affordability, absence of secondary pollution, and mild reaction conditions (temperature and pressure)^25,26^. Many researchers have focused on studying photocatalytic materials such as CuO, ZnO, TiO_2_, and WO_3_ for remediation of environmental pollutants^27–29^. Among these materials, zinc oxide (ZnO) has gained significant attention in the decontamination of pollutants because of its excellent photocatalytic activity, high chemical stability, abundance, low cost, and ease of large-scale preparation with various formes^30^. However, there are two main limitations that hinder the extensive use of ZnO for antibiotic elimination. First, ZnO cannot effectively absorb visible light from sunlight because of its widespread bandgap (3.37 eV)^31,32^. Second, the poor separation of electron– hole pairs and their fast recombination rates impede the generation of reactive oxygen species required for the oxidation of organic compounds^33,34^. To overcome these drawbacks, various techniques have been utilized to enhance the mineralization of contaminants. These methods encompass the incorporation of metals and non-metals, as well as coupling with other semiconductor materials^35^. One approach is the formation of ZnO-based nanocomposites with narrow bandgap semiconductors such as CoFe_2_O_4_, Bi_2_MoO_6_, WO_3_, Fe_3_O_4_, CuO, and InO_3_. This strategy broadens the absorption of solar energy and prolongs the lifetime of charge carriers^36–38^. Cupric oxide (CuO), which possesses advantages such as high stability, affordability, a p-type narrow bandgap (1.2–1.8 eV), and non-toxicity, is considered a promising material for constructing heterostructured p–n nanocomposites with ZnO^39^. CuO presents a promising potential for forming heterostructured p–n nanocomposites when combined with ZnO. CuO possesses several advantages that make it suitable for this application. First, it exhibits high stability, which is crucial for long-term use in environmental remediation processes. In addition, CuO is relatively inexpensive, making it cost-effective for large-scale applications^40,41^.

With magnetic separation characteristics inherent in the entire catalyst, Fe_3_O_4_ delivered electrons to CuO nanoparticles positioned on the Fe_3_O_4_/CuO surface. This has the potential to enhance the catalytic effectiveness of CuO^42,43^. The SiO_2_ shell, featuring perpendicular pore conduits, served a dual purpose. It acted as a physical barrier, preventing the clumping and leakage of Fe_3_O_4_-CuO nanoparticles. Simultaneously, it facilitated the transfer of substances during the catalytic reaction, thereby enhancing catalytic efficiency. Possessing a distinctive nanostructure and a range of functionalities, the composite emerges as an exceptionally efficient, economically viable, and long-lasting catalyst. It demonstrates proficiency in magnetic separation and showcases notable reusability^43,44^. The integration of magnetic materials, like Fe_3_O_4_ nanoparticles, into ZnO/CuO composites is anticipated to boost the efficiency of photocatalysis.

The main objective of our research was to create and produce a novel and unique core–shell thin film to serve as a visible-light-driven photocatalyst. This particular thin film effectively aided in the degradation of AMX, a common antibiotic, in aqueous media. In this research, we developed a SDPR-deposited FSNCZ CS thin-film photocatalyst for the decomposition of amoxicillin (AMX). The spin coating technique was applied to deposit the thin film onto a spinning ceramic disc. The impact of parameters such as pH, initial AMX concentration, rotational speed, solution flow rate, and illumination time were studied and optimized. Finally, the durability and reusability of the FSNCZ CS catalyst film were appraised by conducting repeated experiments under the same conditions.

## Materials and methods

The SDPR comprises a spinning disc with a diameter of 20 cm formed into a cylinder. The disc serves as a platform for depositing the Fe_3_O_4_@SiO_2_-NH_2_@CuO/ZnO nanostructure catalyst. To facilitate the reaction, the disc was exposed to irradiation from a blue LED lamp positioned above it. Throughout all experiments, the distance between the light source and the disc surface remained constant, and the light intensity emitted by the blue LED lamp was maintained at 13 mW/cm^2^. In conducting photocatalytic experiments, the pH level (3–7), AMX initial concentration (10–30 mg/L), solution flow rate (0.3–0.9 L/min), and rotation speed (150–350 rpm) were investigated.

### Chemical reagents used in synthesis and experiments

Purchased from Merck company, Germany, were various chemicals, including copper nitrate trihydrate (Cu(NO_3_)_2_.3H_2_O), zinc nitrate hexahydrate (Zn(NO_3_)_2_.6H_2_O), ferrous chloride tetrahydrate (FeCl_2_∙4H_2_O), ferric chloride hexahydrate (FeCl_3_∙6H_2_O), tetraethyl orthosilicate (TEOS), aminopropyl triethoxysilane (APTES), ammonium hydroxide (NH_3_OH), and additional reagents and solvents. No further purification was conducted, and all reagents and solutions were utilized in their original, as-received state.

### Assembly of the SDPR

Utilizing a SDPR depicted in Fig. S1 facilitated the photodecomposition of AMX in aqueous solutions. The operational mechanism this system has been expounded upon in prior publications^45^. In the SDPR, decomposition of AMX occurred when exposed to visible light by utilizing a thin film photocatalyst composed of FSNCZ CS nanostructure applied onto a rotating ceramic disc. Enclosed within a cylindrical glass chamber, the photocatalytic reactor showcased a ceramic disc, adapted for rotation, and coated with a photocatalyst, illuminated by blue LED lamps. The AMX solution, initially stored in a tank, was subsequently dispensed onto the central region of the disc through a distributor equipped with five holes. The centrifugal force generated by the rotating disc propelled the AMX solution toward the periphery, resulting in the formation of thinner solution films and fine droplets. Enhancing the mass transfer coefficient resulted in a notable reduction in the size of the reactor, distinguishing it from conventional photocatalytic reactors^46^. Providing illumination, a blue LED lamp situated above the stationary enclosure functioned as the light source. Incorporating a control box system, the SDPR allowed for the adjustment of the disc’s rotational speed, activation or deactivation of the blue LED lamp, and control over the pump (for air and water), motor, and mixer functions. The process of photocatalytic decomposition occurred within a spinning disc reactor exposed to visible LED light. To eliminate excess light, the Pyrex glass reactor, cylindrical, was sealed with aluminum foil. Measurement of pollutant concentrations employed a UV–Vis spectrophotometer (JASCO, V 730), while pH levels were regulated using a pH meter of the 827 pH Lab-metrohm type.

### Synthesis of FSNCZ CS

**Figure 1:**
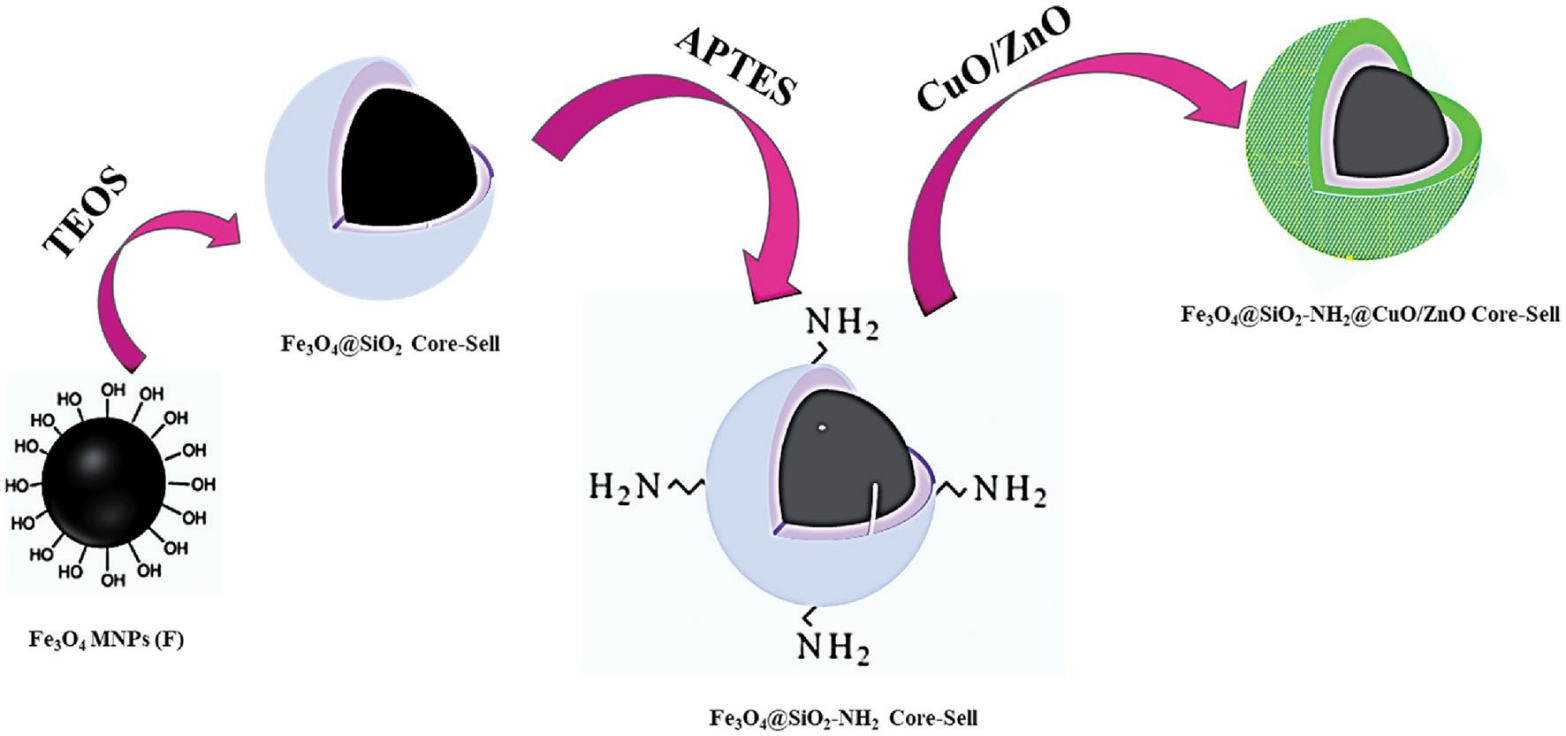
Synthesis steps of FSNCZ CS nanostructure.

The FSNCZ CS nanostructure was synthesized in four sequential steps. First, Fe_3_O_4_ magnetic nanoparticles were prepared by dissolving FeCl_3_∙6H_2_O (3.1 g) and FeCl_2_∙4H_2_O (1.15 g) in 25 mL of deionized water (DI water) at 90 °C for 10 min. A solution of 15 mL NH_3_OH was gradually added until the solution turned black. Using an external magnet, the generated suspended solid was isolated, then washed with DI water and ethanol. It underwent drying in an oven at 60 °C for 12 h. Following this, the Stober method was implemented to produce Fe_3_O_4_@SiO_2_. In this process, 0.5 g of Fe_3_O_4_ obtained from the previous step was mixed in a solution of 50 mL methanol and 8 mL DI water with the assistance of a magnetic stirrer. To this mixture, 5 mL of NH_3_OH solution and 1.5 mL of TEOS were added and stirred for 24 h. After separation using an external magnet, the resulting product underwent washing with both DI water and HCl. Subsequently, it was dried in an oven at 70 °C for a duration of 8 h. In the next step, a combination of 0.5 g Fe_3_O_4_@SiO_2_ core–shell (FSN CZ) in 20 mL methanol was stirred for 15 min. To complete the synthesis of FSN CS, 1.3 mL of APTES was introduced to the Fe_3_O_4_@SiO_2_ core–shell solution under reflux conditions and magnetic stirring at 65 °C for 24 h. The resulting product was separated using an external magnet and dried in an oven at 70 °C for 10 h. NH_2_ functional groups were utilized to bond CuO and ZnO to the FSN CS nanostructure, thereby modifying the surface structure. Finally, the hydrothermal method was utilized to produce the FSNCZ CS nanostructure. In this stage, Cu(NO_3_)_2_∙3H_2_O (1.35 g) and Zn(NO_3_)_2_∙6H_2_O (1.2 g) were dissolved in 50 mL of deionized water, while 0.25 g of FSN nanostructure was dissolved in another 50 mL of DI water. Subsequently, 10 mL of NH_3_OH was added dropwise to the mixed solution of the two compounds, which underwent ultrasonic treatment for 24 h. Using an external magnet, the resultant product underwent separation, followed by washing with DI water and ethanol. Subsequently, it was underwent drying at 60 °C for a duration of 12 h (refer to Fig. 1).

### Immobilization of the FSNCZ CS thin film on the disc

Employing the sol–gel spin coating technique detailed in previous studies^47^, the immobilization of the FSNCZ CS thin film took place on a ceramic disc. Before the deposition of the catalytic film, the ceramic disc substrate was subjected to a cleaning process according to previous studies^47^. In the preparation of the solution, 0.125 g of PVA in 12.5 ml of DI water underwent heating in a sealed container at 80 °C for 3 h to ensure full swelling. Simultaneously, 0.5 g of the synthesized FSNCZ CS powder underwent dissolution in 25 ml DI water, forming a homogeneous solution. Through ultrasonication lasting 1 h, the FSNCZ CS and PVA solutions were meticulously treated until achieving a uniform solution. This resulting solution became instrumental in the deposition of the disc through spin coating, executed at 700 rpm for 60 s. The deposition process comprised applying three layers onto the disc, with each layer undergoing drying at 60 °C for 15 min before the subsequent deposition. Eventually, the coated disc underwent drying at 250 °C for 1 h, marking the completion of the entire process.

### Analytical techniques of synthesized composites

The synthesized materials were characterized using several analytical techniques: FESEM, VSM, XRD, AFM, DRS, EDX, and TEM. The surface characteristics of the materials were examined through FESEM analysis performed on MIRA3 and Zeiss devices (FE-SEM TESCAN MIRA3). VSM analysis determined the magnetic properties using an MDK device (VSM, MDK VSM, Iran). For XRD analysis, the Rigaku Ultima IV device (XRD, Rigaku Ultima IV, Japan) was employed to capture the XRD patterns of the synthesized materials. Additionally, structural characteristics of the synthesized materials underwent examination through TEM analysis, performed using an EM208S device (TEM, Philips EM 208S).

## Results and discussion

### Characterization

**Figure 2:**
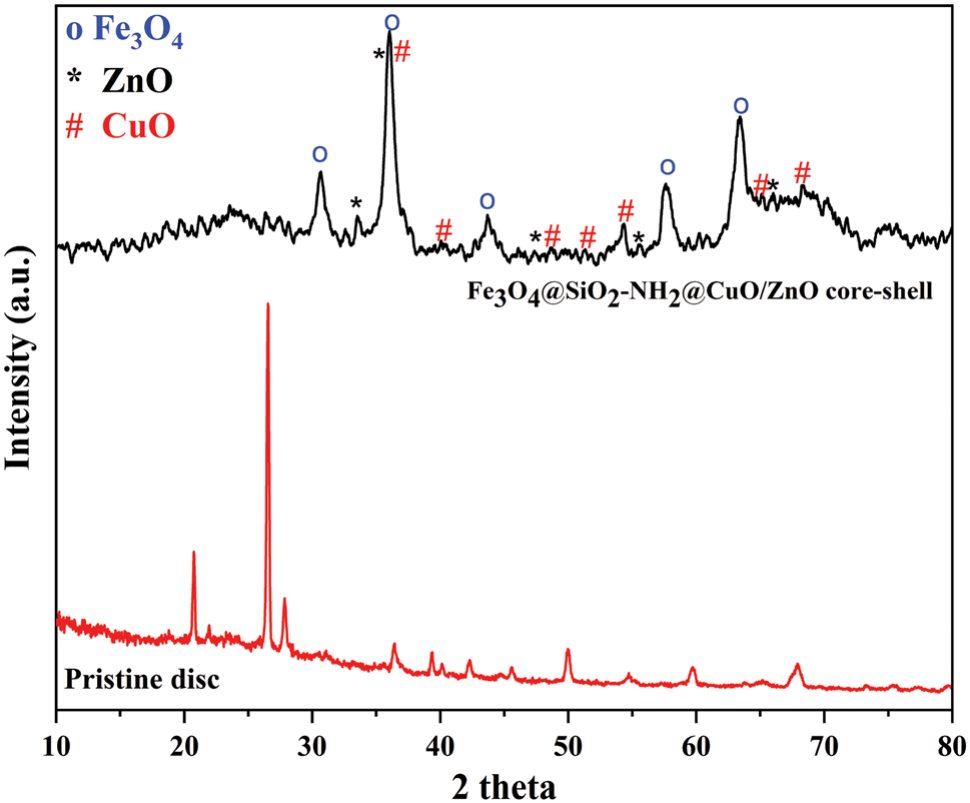
XRD spectra of pristine disc and FSNCZ CS thin film.

XRD technology was used to examine the composition and crystallinity of the obtained samples. The XRD pattern displayed in Fig. S2 indicates that all identified peaks match magnetite Fe_3_O_4_ based on the standard data (JCPDS No. 088-0315) for magnetite. No impurities were detected. The Fe_3_O_4_ microspheres exhibited five prominent diffraction peaks at angles of 30.36°, 35.82°, 43.47°, 57.49°, and 63.1°, corresponding to the (2 2 0), (3 1 1), (4 0 0), (5 1 1), and (4 4 0) planes, respectively, of the face-centered cubic structure of magnetite. In comparison to Fig. S2-a, the introduction of a SiO_2_ layer results in a marginal reduction in intensity within the XRD pattern of the Fe_3_O_4_@SiO_2_ (Fig. S2-b). This reduction could be attributed to the existence of amorphous silica. The crystal structure of Fe_3_O_4_ nanoparticles has not changed after the coating of SiO_2_ and NH_2_ around it. In addition, the coating of SiO_2_ and NH_2_ around Fe_3_O_4_ nanoparticles is amorphous and not crystalline (Fig. S2-c)^48^. After introducing zinc ZnO and CuO grains onto the surface of the FSN CS nanostructure (Fig. S2-c), a multitude of new peaks becomes apparent at 2θ values of 31.29, 34.16, 47.54, 55.76, 68.28, and 68.86. These peaks are assignable to the ZnO hexagonal wurtzite phase and, respectively, at 2θ values of 33.42, 37.8, 39.45, 48.95, and 69.8, they correspond to the monoclinic copper oxide^49^. Furthermore, a comparison between the two XRD patterns—one from the pristine disc and the other from the FSNCZ CS thin film in Fig. 2—reveals the lack of any additional peak in the core–shell film associated with crystalline phases formed by the pristine disc.

Detailed insights into the surface morphology and size distribution of the synthesized magnetic nanomaterials are provided by FESEM, illustrated in Fig. S3. The Fe_3_O_4_@SiO_2_ core–shell nanostructure manifests a roughly granular and spherical shape within the size range of 66–97 nm (Fig. S3-a). Similarly, the microscopy image of the FSN CS nanostructure depicts uniform particles ranging from 56 to 94 nm (Fig. S3-b). In the instance of the FSNCZ CS nanostructure, FESEM images captured indicate surface modifications of the nanoparticles, with the size distribution spanning from 62–96 nm (Fig. S3-c). Therefore, FESEM images can be used to identify the structure of the nanocatalyst. Transmission electron microscopy (TEM) was applied to scrutinize the composition, structure, size, and morphology of the FSNCZ CS nanostructure.

**Figure 3:**
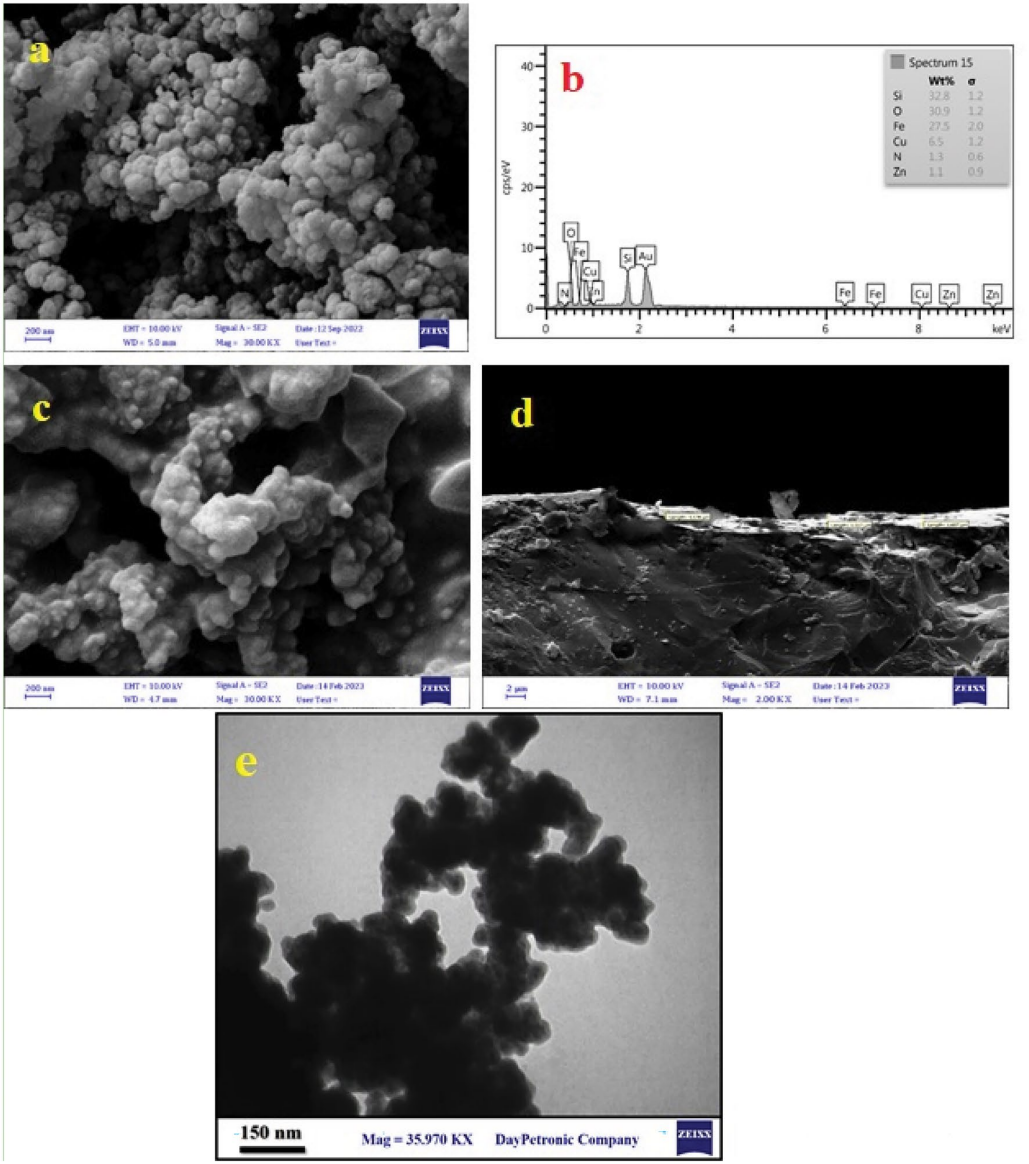
FESEM image of FSNCZ CS nanostructure: EDAX (**a**,**b**) and top-of-view and cross-sectional views of the core–shell film (**c**,**d**) and TEM image of Fe_3_O_4_@SiO_2_-NH_2_@CuO/ZnO Core–Shell nanostructure (**e**).

Figure 3 presents the results of FESEM analyses conducted on the surface of ceramic disc coated with the FSNCZ CS film. Verification of the presence of spherical structures distributed across the disc surface, characteristic of the FSNCZ CS, is evident in the FE-SEM images exhibited in Fig. 3a. Figure 3-b showcases the EDX spectra of the immobilized thin film on the disc, providing a detailed quantitative elemental analysis. The average thickness of the FSNCZ CS film, approximately 2.83 μm, is indicated in Fig. 3-c,d. This analysis underscores that the FSNCZ CS nanostructure film contributes to the enhancement of both mass transfer rate and pollutant adsorption, resulting in improved photocatalytic activity. TEM image Fig. 3e shows the morphological images of FSNCZ CS nanostructure with an average particle size of 38.77 nm. The TEM image depicts that the Fe_3_O_4_ nanoparticles inside the core–shell structure are almost spherical and dark in shape, and the average size is about 12.67 nm, while the silica shell is somewhat gray. The image shows that the CuO and ZnO nanoparticles are covered in a uniform layer on the silica surface. These observations strongly indicate the successful formation of FSNCZ CS nanostructure.

**Figure 4:**
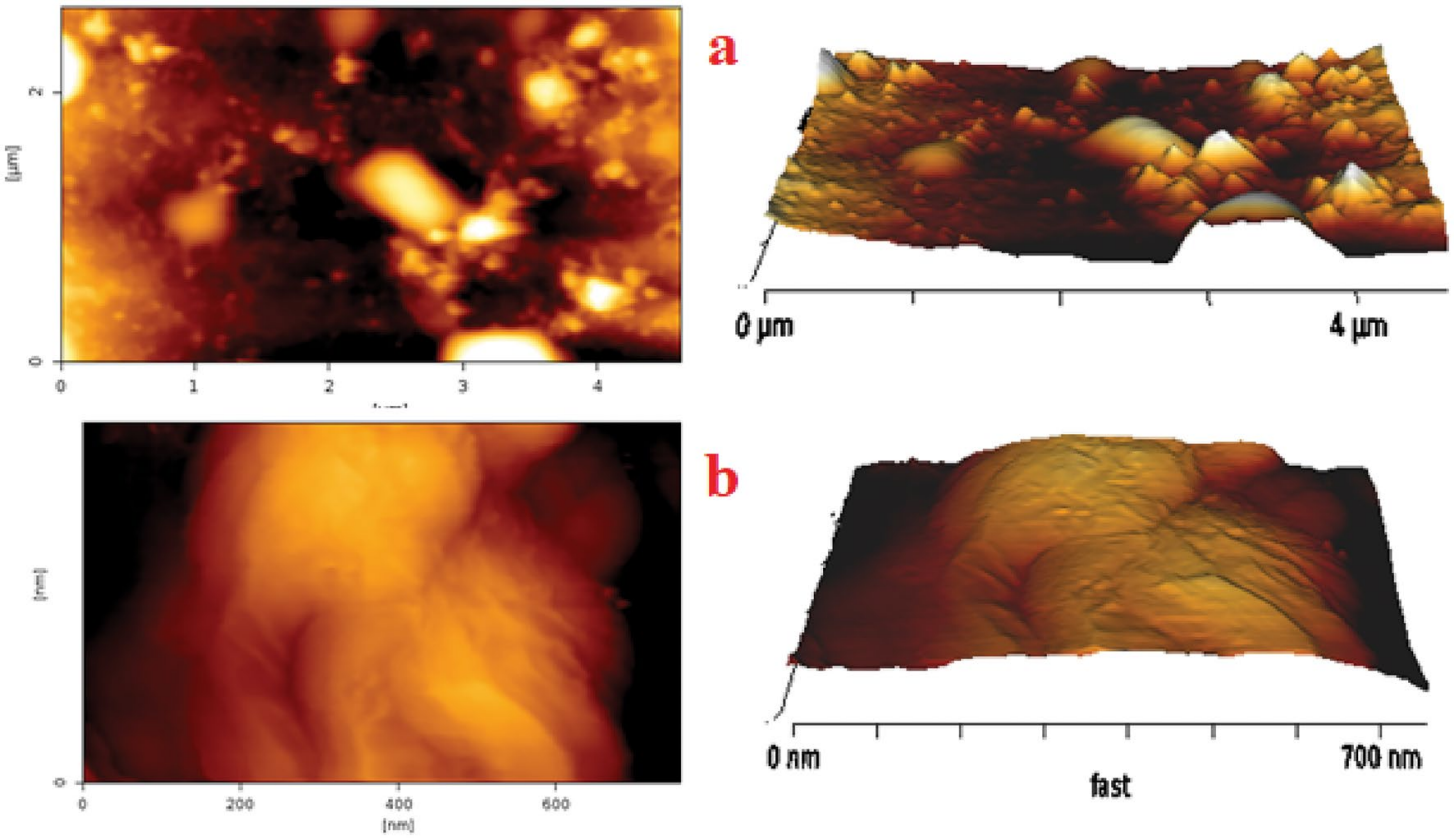
2D and 3D AFM images of (**a**) the pristine disc and (**b**) FSNCZ CS film.

The assessment of the surface topology and roughness of the FSNCZ CS thin film was conducted using AFM analysis (refer to Fig. 4). Percolated nanostructures were observed in the fabricated thin films. Subsequent to the addition of FSNCZ CS, a distinct increase in surface roughness was noted, aligning with the observations from FESEM images. This uniform enhancement in surface roughness has the potential to elevate the rates of mass transfer, AMX adsorption, and light distribution, ultimately contributing to an improved efficiency in photodecomposition.

**Figure 5:**
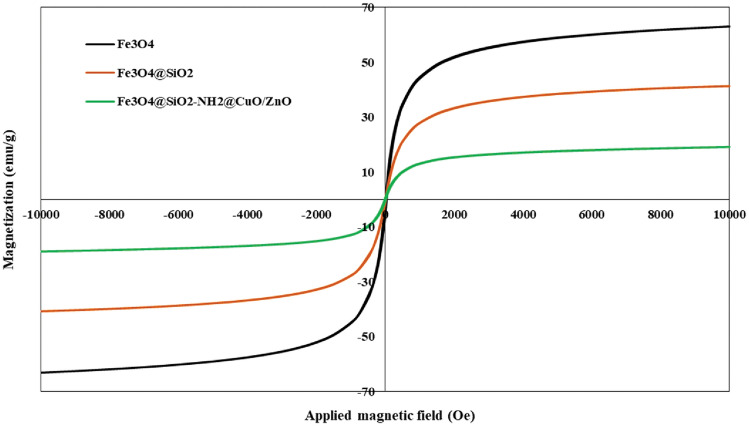
Magnetic saturation of the synthesized samples of Fe_3_O_4_, Fe_3_O_4_@SiO_2_, and FSNCZ CS.

VSM analysis was conducted to investigate the magnetic features of Fe_3_O_4_, Fe_3_O_4_@SiO_2_, and FSNCZ CS samples (Fig. 5). The results, depicted in Fig. 5, show that Fe_3_O_4_ MNPs exhibit superparamagnetic behavior with a magnetization of approximately 63 emu/g at an employed field of 10,000 Oe. When amorphous silica is incorporated as a shell surrounding Fe_3_O_4_, the resulting Fe_3_O_4_@SiO_2_ sample exhibits a reduced magnetization of approximately 41.32 emu/g under the same applied field. This decrease in magnetization can be related to the existence of a silica shell around Fe_3_O_4_. The saturation magnetization value for FSNCZ CS was found to be 19.12 emu/g. These results indicate that upon coating Fe_3_O_4_ with SiO_2_, ZnO, and CuO, saturation magnetization noticeably decreased^50^. However, the resulting products retained sufficient magnetization to enable magnetic separation using a magnetic field. This feature facilitates the separation of photocatalytic materials from the treated solution.

The technique of diffuse reflectance spectroscopy (DRS) is employed to determine the optical transitions. Equation (1)expresses the relationship between the band gap (Eg) and the absorption coefficient (α)^51^.

1 $$\left( {\alpha {\text{h}}\upsilon } \right)^{{2}} = \beta \left( {{\text{h}} - {\text{E}}_{{\text{g}}} } \right)$$

Within this equation, the proportionality constant (β), and the photon frequency (ʋ) are denoted. The bandgap can be estimated by plotting (αhʋ)^2^ in terms of h based on the obtained experimental data. The direct bandgap of the sample is obtained by determining the width from the origin of a straight line at α = 0.

**Figure 6:**
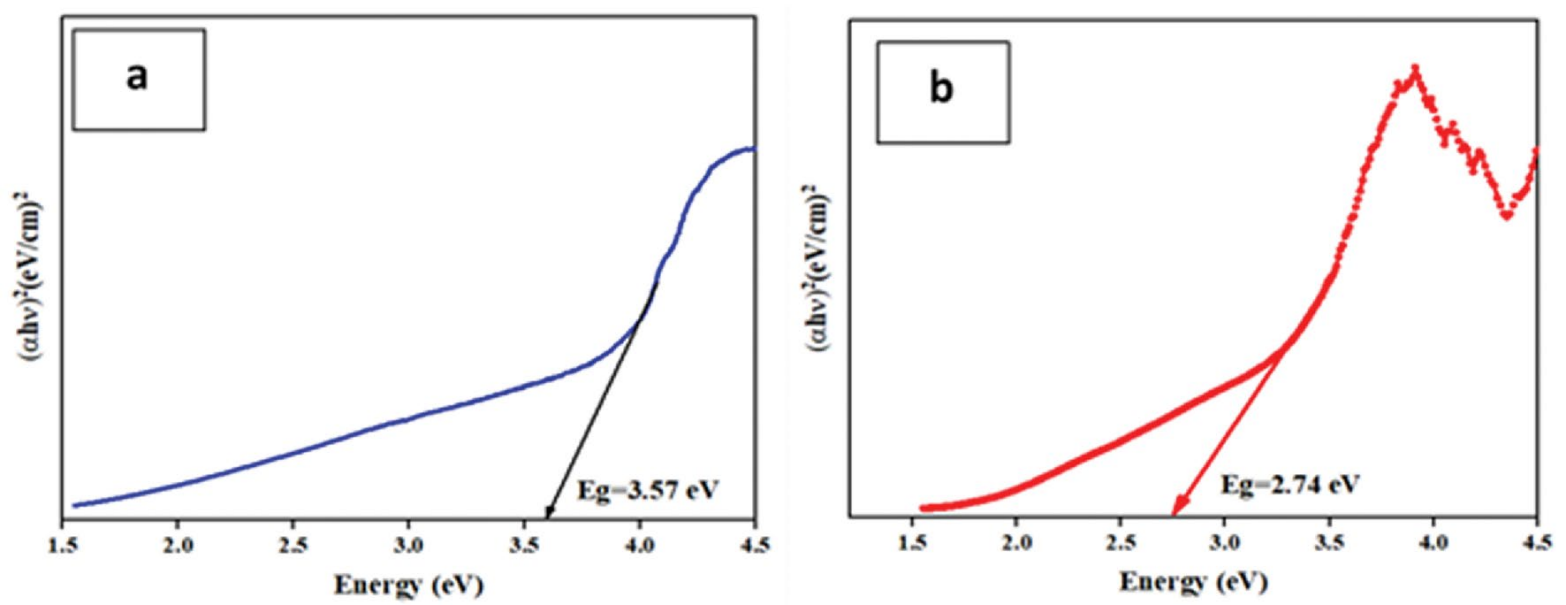
UV–Vis DRS spectra of (**a**) FSN CS and (**b**) FSNCZ CS.

The UV–Vis DRS patterns were employed to analyze the optical characteristics of the FSN CS and FSNCZ CS nanostructures. Within the standard UV–Vis absorption range, the absorption peaks of the as-prepared FSN CS and FSNCZ CS nanostructures were identified, as illustrated in Fig. 6. The band gap energy values for the FSN CS and FSNCZ CS samples were determined, resulting in values of 3.57 and 2.74 eV, respectively. In comparison to the FSN CS sample, the FSNCZ CS sample exhibited heightened absorption in the visible light region. The observed enhancement can be ascribed to the increased CuO concentration in the synthesized sample. Unlike regular materials with continuous energy bands, nanostructures exhibit discrete energy levels due to their nanodimensionality. As the size of nanoparticles decreases, their band gap increases, illustrating the "quantum size effect." Therefore, the modification in the band gap is influenced by both the quantum size impact and the surface impact of FSN CS and FSNCZ CS nanostructures^52,53^.

### Optimization of the photocatalytic decomposition process

To optimize operational parameters, the one factor at a time (OFAT) method was used. In this study, four factors, pH (3–7), initial concentration of amoxicillin (10–30 mg/L), solution flow rate (0.3–0.9 L/min), and disk rotation speed (150–350 rpm), were investigated during the irradiation time of 0–75 min.

**Figure 7:**
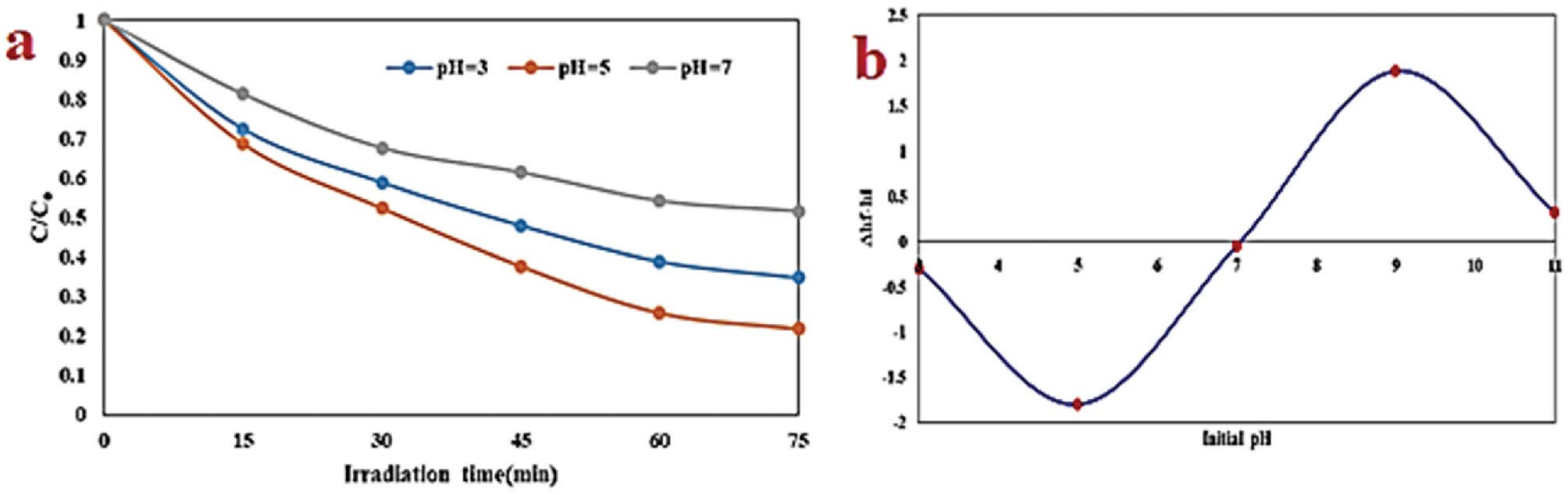
Impact of pH on AMX decomposition (starting AMX concentration of 20 mg/L, flow rate of 0.6 L/min, rotational speed of 150 rpm, and irradiation time of 75 min) (**a**) and pHpzc diagram (**b**).

The pH level of the solution plays a substantial task in influencing the decomposition of AMX. This influence is the result of diverse factors, encompassing the surface charge of the catalytic film, the ionization of AMX, and the configuration of functional groups on the sites of the photocatalyst^54^. To evaluate the efficiency of AMX decomposition, experiments were conducted at pH levels ranging from 3 to 7, solution flow rates of 0.3–0.9 L/min, rotational speeds of 150–350 rpm, initial AMX concentrations of 10–30 mg/L, and illumination times of 75 min. The reaction was performed using the FSNCZ CS thin film under visible LED light. Figure 7 illustrates the decomposition efficiency of AMX against a pH range of 3–7. As depicted in Fig. 7-a, there is a noticeable increase in the efficiency of AMX decomposition from pH 3 to 5, followed by a decrease from pH 5 to 7. In this study, pH 5 was identified as the optimal pH level at which the FSNCZ CS photocatalyst film achieves a maximum efficiency of 78.3%. The varying performance efficiencies of AMX and the photocatalyst at different pH values can be attributed to their distinct properties. The pH zero point charge (pHzpc) of the FSNCZ CS photocatalyst was determined based on previous studies^55^ and found to be 7.1. As illustrated in Fig. 7-b, the photocatalyst surface is positively charged at pH < pHzpc and negatively charged at pH > pHzpc. AMX has ionic dissociation constant pKa1 = 2.68) corresponding to carboxyl ionizable functional group^56^. Kanakaraju et al. discovered that AMX is stable toward hydrolysis in the pH range of 5.5–6.5^57^. This process is ascribed to the transformation of carboxyl groups (-COOH) within the molecular structure of amoxicillin into carboxylate (–COO–). Consequently, it results in the adsorption of negatively charged amoxicillin molecules (–COO–) onto the positively charged surface of the FSNCZCS structure. This phenomenon, in turn, contributes to an increase in decomposition efficiency^58^. However, the decrease in decomposition efficiency at alkaline pH levels can be related to repulsive force between the negatively charged surface of the FSNCZ CS structure and the anions for absorption on the active sites of the adsorbent surface^59^. It is also shown that the decomposition efficiency reduces in the pH range between 5 and 7. This phenomenon is probably due to the increased formation of hydroxide ions (OH–) and strong competition between OH– and –COO– anions in the adsorption of amoxicillin molecules to the active sites of the catalyst surface^60^.

**Figure 8:**
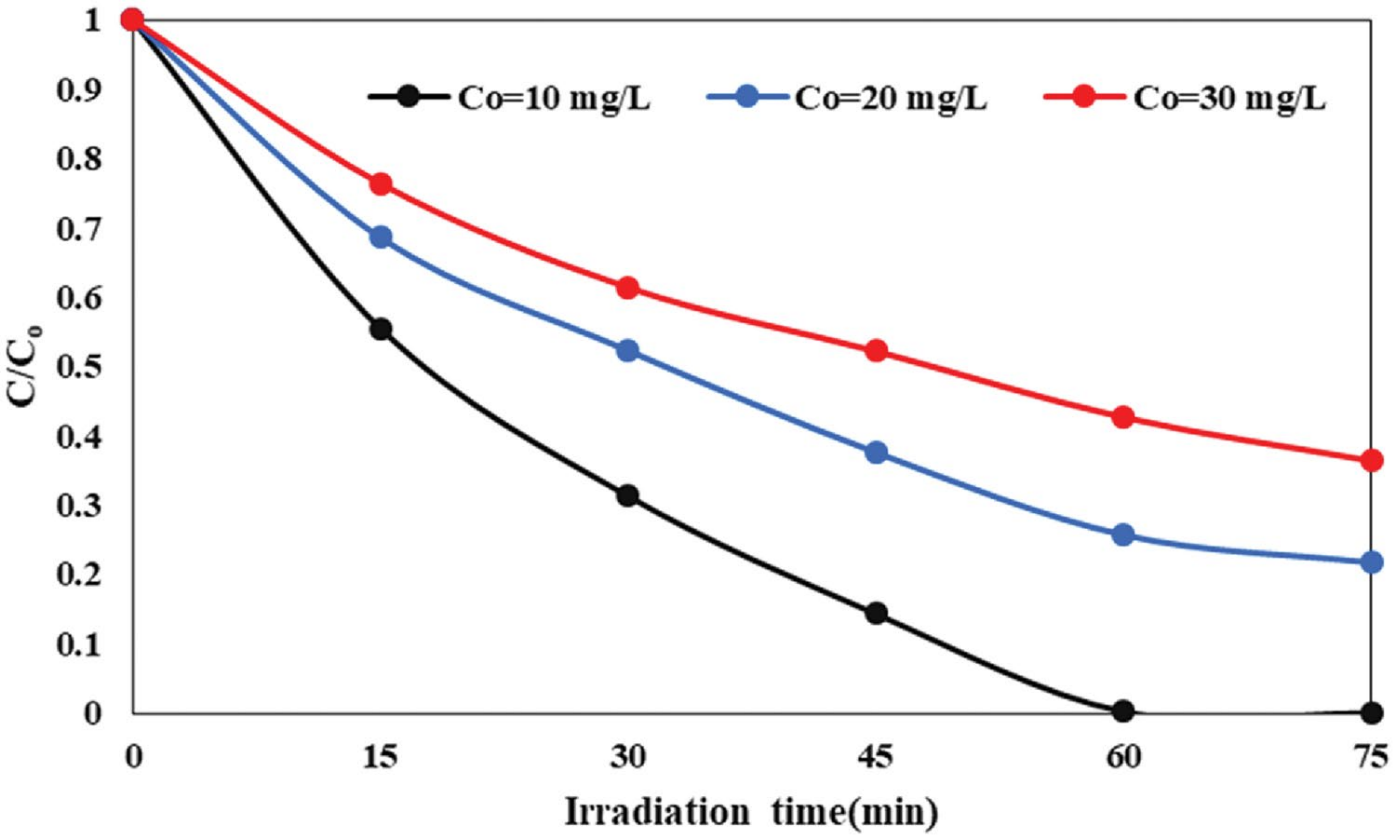
Impact of the initial concentration of AMX on the photodecomposition in the SDPR.

The initial concentration of AMX is pivotal in the photocatalytic decomposition process. The experimental findings depict that the highest efficiency in removing AMX, or the amount of AMX that underwent decomposition, occurred at concentration of 10 mg/L after 60 min of irradiation (Fig. 8). Specifically, at a 60 min irradiation time, 100% of the AMX in the 10 mg/L solution was degraded, whereas at 75 min, 78.3% of the AMX in the 20 mg/L solution was degraded. However, as the initial concentration of AMX raised from 10 to 30 mg/L, there was a reduction in the decomposition rate. The optimal efficiency for AMX decomposition was found to be 78.3% at concentration of 20 mg/L. Nevertheless, as the initial AMX concentration continued to rise beyond 20 mg/L, the decomposition efficiency gradually decreased. In summary, the degradation efficiency of the AMX solution is greater at lower initial concentrations, influenced by factors such as the photocatalyst’s surface area, availability of oxidants, competition among AMX molecules, and a reduction in the light intensity achieving the catalyst surface. At higher concentrations, more light is dispersed by the solution, leading to a reduction in electron–hole pair generation and a subsequent decrease in decomposition efficiency. Additionally, higher concentrations of AMX necessitate more active oxidant species for decomposition, and an excess concentration on the catalyst surface impedes light absorption and may hinder access to the catalyst surface. Compounds generated as intermediates throughout the decomposition process can also hinder the advancement of the photocatalytic reaction.

**Figure 9:**
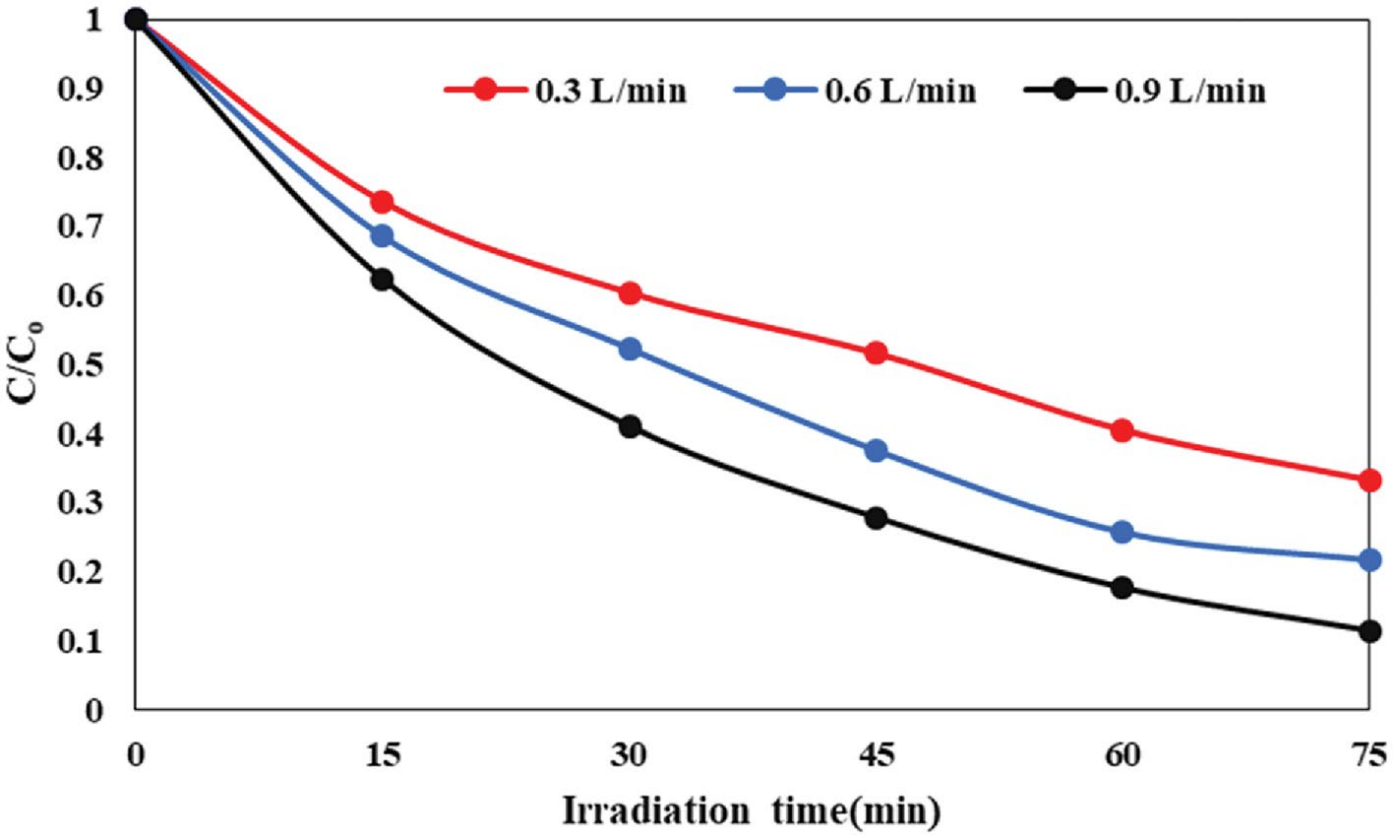
Impact of flow rate on the decomposition of AMX in the SDPR.

The study investigated the impact of flow rate on AMX decomposition efficiency, covering a range from 0.3 to 0.9 L/min. This examination was conducted within specific conditions, including pH = 5, an initial AMX concentration of 20 mg/L, a rotation speed of 150 rpm, and an irradiation time of 75 min, employing a thin film photocatalyst. As exhibited in Fig. 9, the decomposition efficiency of AMX increased (from 66.7 to 78.3%) with the rise in flow rate from 0.3 to 0.9 L/min. Two factors contribute to the variation in decomposition efficiency with changing flow rates. Firstly, at a flow rate of 0.3 L/min, there is a potential breakdown of the liquid film and inappropriate distribution of fluid flow on the disc, reducing the mixing ability of the fluid film and subsequently decreasing degradation efficiency at this flow rate. Secondly, the augmentation of the flow rate from 0.3 to 0.9 L/min amplifies the interaction between the FSNCZ CS photocatalyst particles and AMX molecules. This enhancement leads to an improved mass transfer rate and decomposition efficiency, reaching an optimum condition at a flow rate of 0.9 L/min^61^.

**Figure 10:**
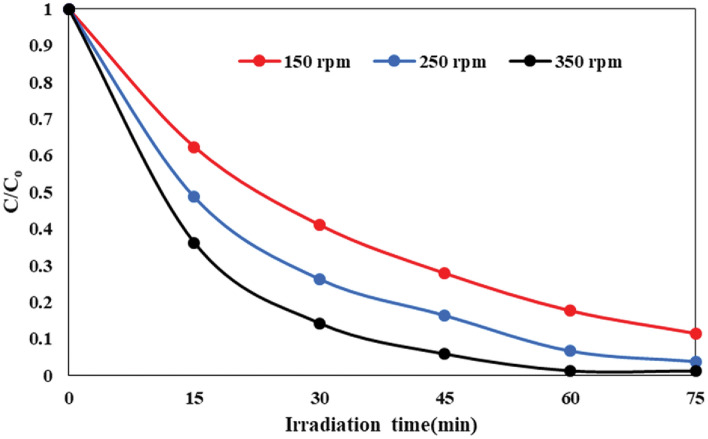
Impact of rotation speed on the decomposition of AMX in the SDPR.

The rotational speed significantly affects the photocatalytic reaction and is considered to be one of the most influential operating parameters (see Fig. 10). This parameter is linked to the mass transfer coefficients of the contaminants and the thickness of the fluid film^45^. Raising the disc rotation speeds from 150 to 350 rpm led to an enhancement in the AMX photodecomposition efficiency from 88.5 to 98.7%. The decomposition efficiency under optimal conditions during 60 min of irradiation time was 98.7% at a rotation speed of 350 rpm. Increasing the disc rotational speed from 150 to 350 rpm has shown to significantly improve the degradation efficiency of AMX, and this enhancement can be attributed to two key factors. Firstly, the increase in rotational speed leads to heightened turbulence and mixing within the system. This enhanced agitation plays a crucial role in promoting better contact between the amoxicillin antibiotic molecules and the FSNCZ CS coated on the disc’s surface. The greater turbulence facilitates a more thorough mixing of the reactants, creating a higher interfacial area for chemical reactions to take place. This increased interaction between the AMX molecules and the photocatalyst is essential for accelerating the degradation process. Secondly, the higher rotational speed enables a more rapid mass transfer rate. The agitation caused by the increased speed results in the formation of a thin sheared liquid film and very fine droplets on the disc’s surface. These conditions further amplify the contact area between the AMX molecules and the photocatalyst, facilitating enhanced interaction between the two components. The greater mass transfer rate ensures that the reactants are efficiently transported to the active sites on the photocatalyst, where degradation reactions can occur more effectively^45,62^.

### Comparison of the different processes

**Figure 11:**
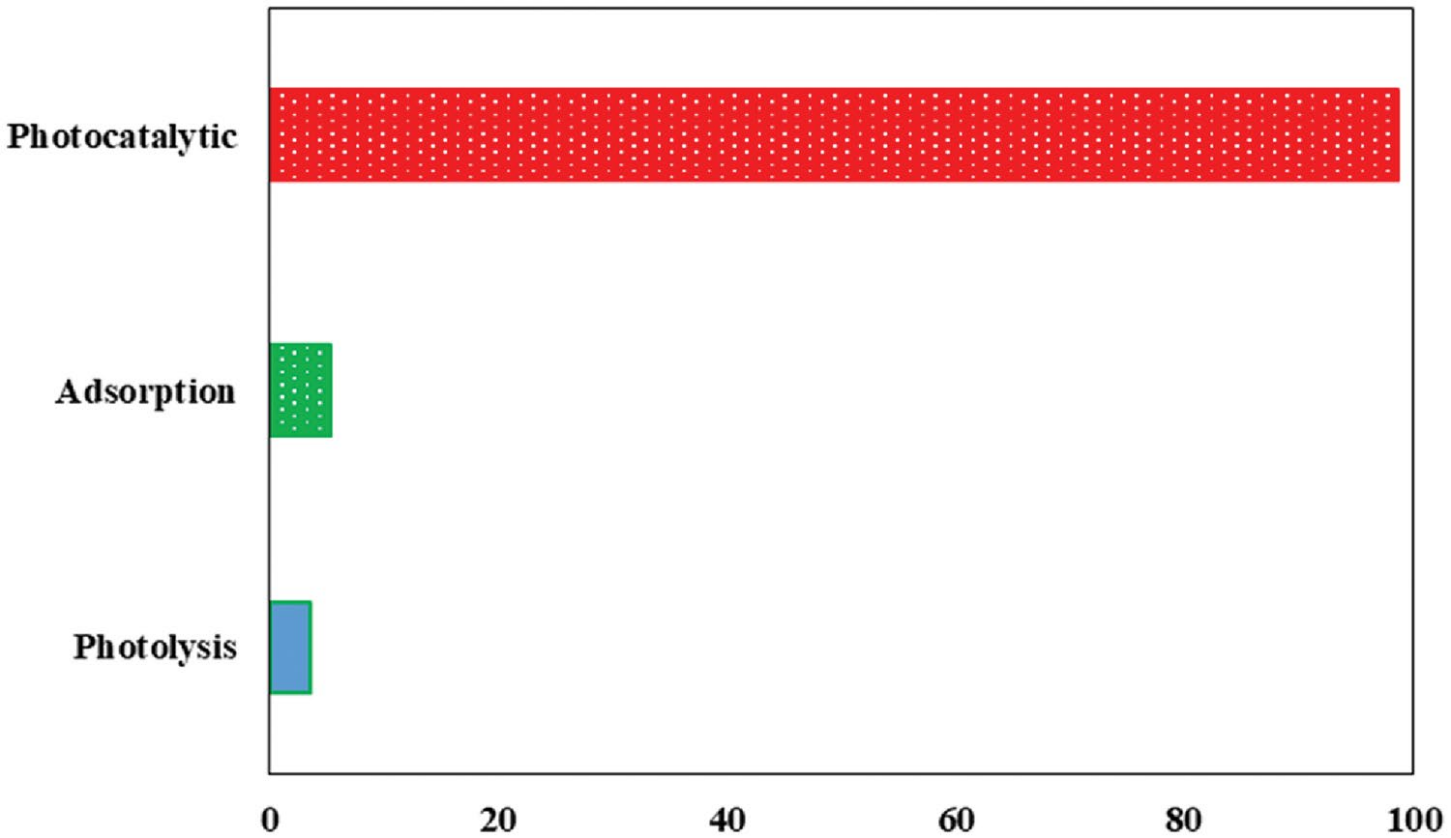
Removal efficiency of AMX for different processes (LED alone, catalyst film alone, and LED/Catalyst film processes) under optimal Conditions.

To assess the efficacy of treating an amoxicillin solution under optimal conditions, the influence of photolysis and adsorption was examined (Fig. 11). In the photolysis process, the AMX solution was subjected to a 30 min test without a catalyst and without exposure to visible light. The lack of oxidizing species is attributed to the inefficient decomposition of AMX under visible LED light in the absence of a catalyst. Therefore, the contribution of photolysis to the decomposition of amoxicillin is minimal. The FSNCZ CS film exhibited weak catalytic activity without exposure to visible LED light since the catalyst requires light to activate its sites. However, upon exposure to visible LED light, the FSNCZ CS film displayed a significant increase in decomposition efficiency. The presence of photons produced electron–hole pairs in the catalyst, leading to the production of free radicals and thereby enhancing the decomposition efficiency.

### Determination of mineralization and biodegradability

**Figure12:**
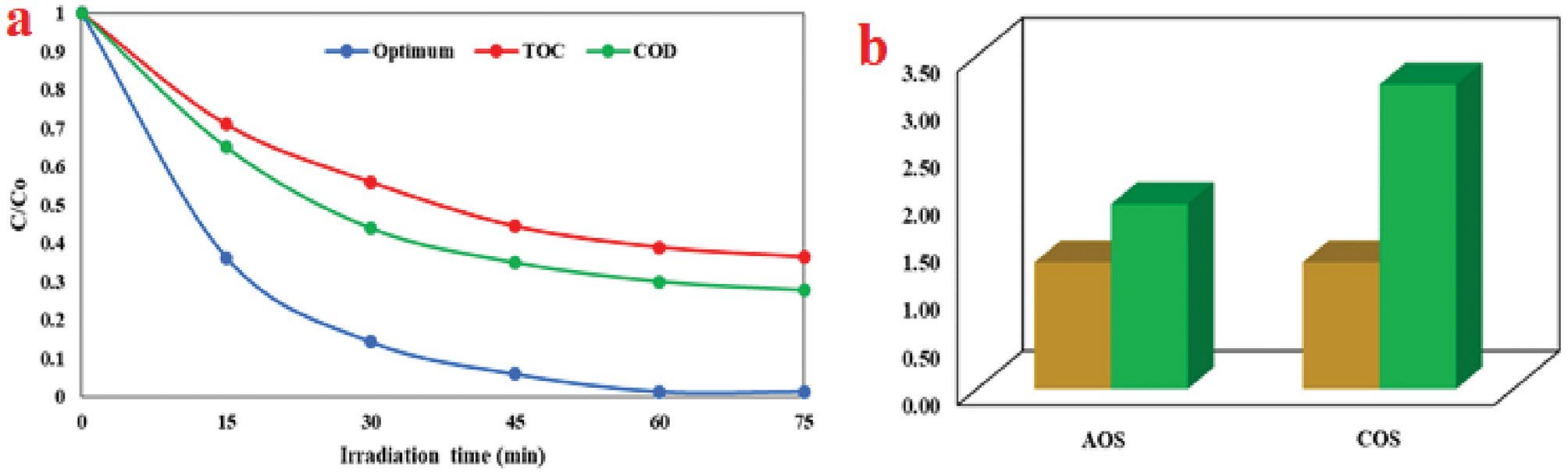
The removal efficiency of COD and TOC (**a**) and biodegradability of AMX (**b**) AOS and COS investigation under optimal conditions.

COD and TOC analyses were employed to examine the mineralization process of AMX using a SDPR under optimum conditions (Fig. 12). The findings depicted that extending the irradiation time enhanced the removal efficiency of COD and TOC by 69.95% and 61.2%, respectively (Fig. 12-a). However, the mineralization rate was less than the removal efficiency of AMX at the optimal reaction time, potentially attributed to the formation of intermediate compound^63,64^. However, as the irradiation time increased, there was a relative improvement in the degree of mineralization, indicating a positive impact of the photocatalytic decomposition process. The average oxidation state (AOS) and carbon oxidation state (COS) indicate the level of mineralization and biodegradability improvement in wastewater. The AOS and COS values can be determined using Eqs. (2) and (3)^65^.

2 $${\text{AOS}}=4-1.5(\frac{{\text{COD}}}{{\text{TOC}}})$$

3 $${\text{COS}}=4-1.5(\frac{{\text{COD}}}{{{\text{TOC}}}_{0}})$$

It shows COD and TOC after photocatalytic decomposition of AMX. TOC_0_ denotes the initial TOC of AMX (mg/L). AOS and COS range from + 4, which represents the most oxidized state of CO_2_, to − 4, which signifies the most reduced state of carbon represented by CH_4_^66^. AOS is a widely used parameter in studying the biodegradability of wastewater. It provides information about the oxidation state of organic compounds in water solutions. In a similar vein, the biodegradability of organic compounds in wastewater is evaluated through COS. In this particular investigation, the initial values for AOS and COS were computed as 1.33 (Fig. 12-b). Nevertheless, following the photocatalytic process, there was a notable elevation in the values of AOS and COS, reaching 1.94 and 3.2, respectively. These increased values suggest a heightened biodegradability of the organic content in the treated samples. Thus, it can be reasonably inferred that the quantity of non-degradable materials witnessed a decrease after the photocatalytic process.

### Experiments of scavenging radicals

**Figure 13:**
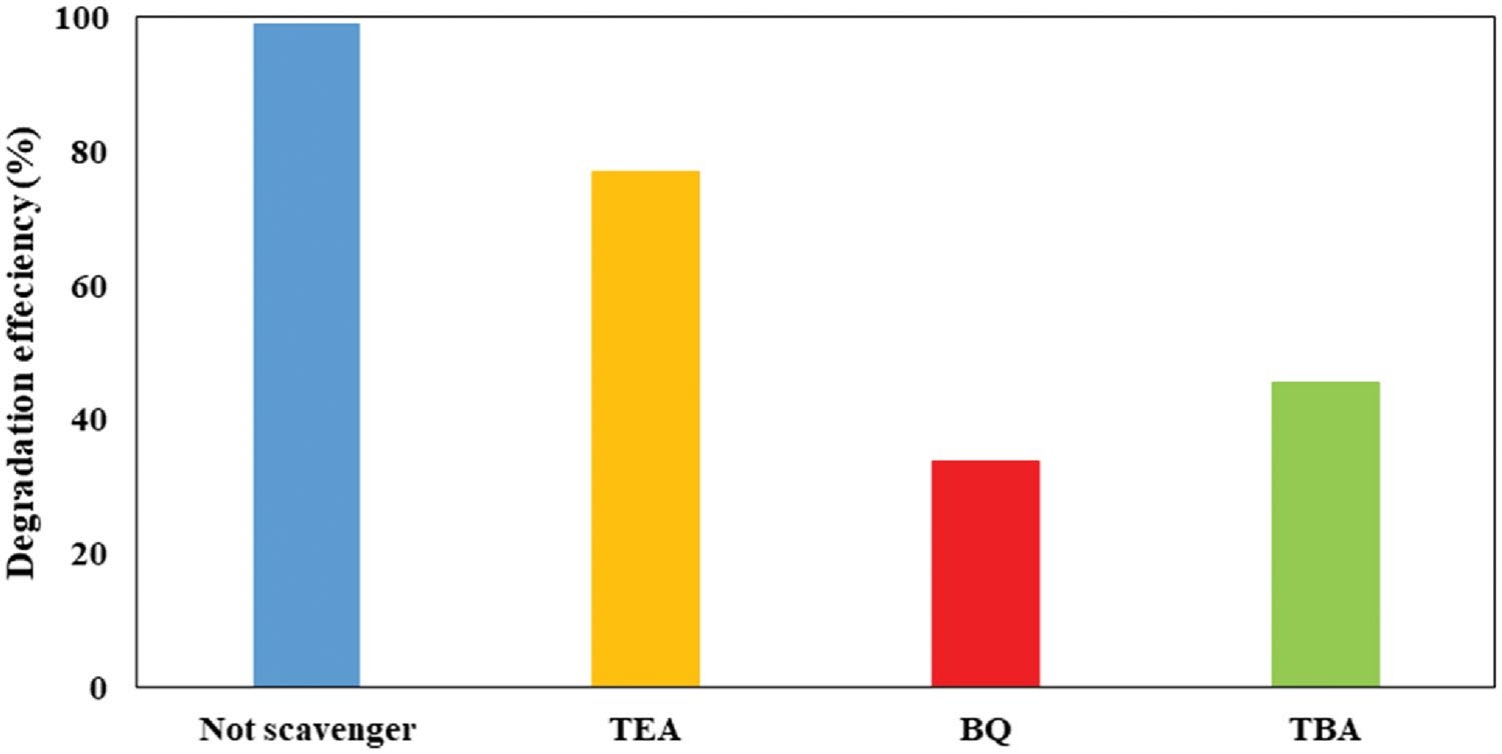
Effect of radical scavenger under optimal conditions.

In order to unravel the mechanism governing the photocatalytic decomposition of AMX under optimal experimental conditions, researchers delved into the examination of the reactive oxygen species produced throughout the photodecomposition process within the SDPR. They conducted experiments employing different scavengers (tert-butanol—TBA, benzoquinone—BQ, and triethanolamine—TEA) to explore the inhibition of hydroxyl radicals (·OH), superoxide radicals (·O_2_^−^), and active holes (h^+^), which are the major active species involved^67,68^. The presented findings in Fig. 13 distinctly outline the specific functions carried out by reactive oxygen species during the photocatalytic process when utilizing the FSNCZ CS film. Significant disparities in the AMX decomposition rate were evident upon the addition of TBA, BQ, and TEA, in contrast to the control group without scavengers. It is noteworthy that the addition of these scavengers adversely affected the photocatalytic activity of the FSNCZ CS film. The researchers determined the reactivity order of reactive oxygen species on FSNCZ CS photocatalysts as follows: BQ > TBA > TEA > no scavengers. The results suggest that BQ depicted the most reactivity towards reactive oxygen species, with TBA and TEA following, and the absence of scavengers showing the least reactivity. Consequently, the main cause of AMX photodecomposition when using the FSNCZ CS film can be attributed to O_2_ and OH radicals, identified as the primary active species. These findings deepen our comprehension of the mechanisms involved in the photocatalytic decomposition process and provide insights into the specific roles of reactive oxygen species in the elimination of AMX^6,47^.

### Durability and reusability of the catalyst film

**Figure 14:**
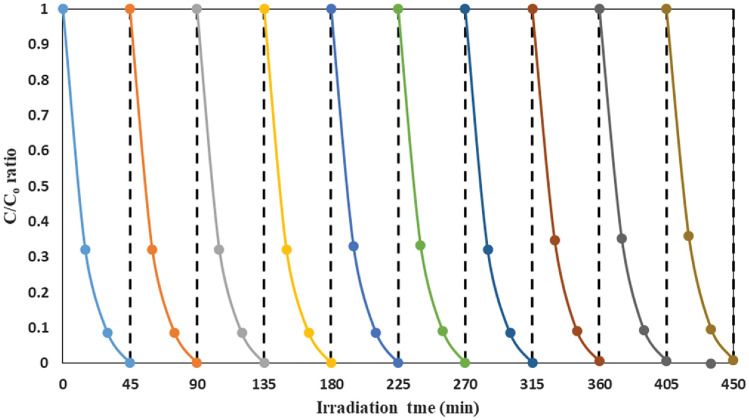
Reusability and durability of FSNCZ CS film as a result of removal of AMX decomposition under optimal conditions.

The evaluation of the reusability and durability of the photocatalyst film constitutes a pivotal aspect in gauging the feasibility and cost-effectiveness of a photocatalytic reactor. To assess the stability of the FSNCZ CS thin film under optimal conditions—including a pH of 5, solution flow rate of 0.9 L/min, initial AMX concentration of 20 mg/L, rotation speed of 350 rpm, and irradiation time of 60 min—ten consecutive decomposition runs were conducted. After each run, the catalyst film was washed and dried. The results shown in Fig. 14 indicate that the photocatalytic activity remained consistent after 10 runs, indicating the excellent reusability of the system. This is a desired quality in practical applications of photocatalytic reactors because it reduces the need for frequent replacement of the catalyst film, thereby lowering the economic cost. The high reliability and proper photocatalytic behavior of the catalyst film make it a promising technology for contaminant decomposition. This suggests that the proposed technology, known as SDPR, has the potential for efficient contaminant decomposition.

### Proposed mechanism of decomposition

**Figure 15:**
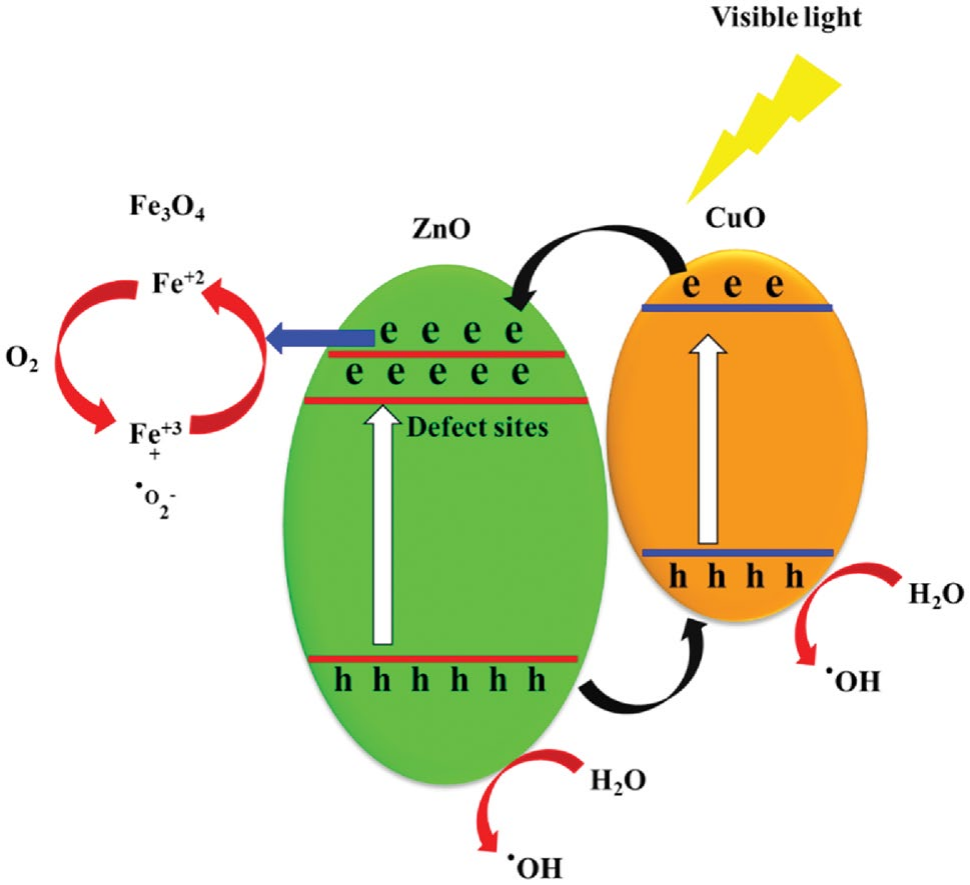
Proposed mechanism of amoxicillin antibiotic decomposition.

In this study, a spinning disc photocatalytic reactor was utilized to decompose the antibiotic amoxicillin using a thin film of nanostructured FSNCZ CS material. CuO, with its narrow band gap, serves as a promising visible light photocatalyst, while ZnO acts as a photocatalyst under UV light. The strategic alignment of band energy levels is crucial for facilitating efficient charge transfer between CuO, ZnO, and Fe_3_O_4_, as illustrated in Fig. 15. When exposed to visible LED light, CuO is activated due to its suitable band gap energy, leading to the generation of electron–hole pairs^69,70^. Meanwhile, under excitation, electrons in the valence band of ZnO can transition to localized defect states, such as oxygen vacancies, with lower energy compared to the conduction band of ZnO. Initially, the formation of ZnO results in a partially reduced oxide with oxygen vacancies as point defects. The depiction in the inner section of Fig. 15 confirms the presence of oxygen vacancies as localized defects within ZnO. Electrons in an excited state from CuO can migrate to the conduction band of ZnO, where they combine with Fe^+3^ to form Fe^+2^ ions. Unstable Fe^+2^ ions interact with O_2_ molecules, leading to the production of ·O_2_ radicals. On the other hand, holes generated from the valence band of ZnO can either migrate to the valence band of CuO or react with water molecules, producing ^·^OH radicals. Additionally, holes in CuO can react with water, resulting in the formation of ^·^OH radicals. The accumulation of electrons by Fe^+3^ ions increases the likelihood of generating holes, which contribute to the creation of ^·^OH radicals that directly target organic pollutants.

## Conclusion

The application of the spin coating technique to immobilize FSNCZ CS thin film catalysts onto the disc surface of a spinning disc photocatalytic reactor proved successful in this investigation. This catalyst film was employed for the photocatalytic decomposition of AMX. The study aimed to investigate the impact of various parameters, including pH, rotational speed, solution flow rate, and initial AMX concentration, during a 75-min irradiation period. The decomposition rate of AMX was found to be minimal when using only the FSNCZ CS thin film nanostructure in SDPR. However, the introduction of the FSNCZ CS thin film nanostructure with LED light significantly enhanced the decomposition rate. At the perfect parameters comprising an irradiation time of 60 min, rotation speed of 350 rpm, flow rate of 0.9 L/min, pH level of 5, and initial AMX concentration of 20 mg/L, the highest level of AMX decomposition reached 98.7%. Finally, the photocatalyst film demonstrated remarkable reusability and durability, as evidenced by consistent and reproducible photodecomposition results over ten consecutive runs under optimal conditions. Based on the outstanding catalytic durability observed in the SDPR system, this film can be considered a viable choice for scaling up the treatment of pharmaceutical manufacturer wastewater.

### Supplementary Information

Supplementary Figures.

## Data Availability

The datasets generated and analyzed during the current study available from the corresponding author on reasonable request.
