# Supplementary material for: Novel nanostructure approach for antibiotic decomposition in a spinning disc photocatalytic reactor
Source: Sci Rep. 2024 May 8;14:10566. doi: 10.1038/s41598-024-61340-8 (PMC11079042; doi:10.1038/s41598-024-61340-8)
Supplement: Supplementary file 1 — Supplementary Figures. [file 41598_2024_61340_MOESM1_ESM.docx]

**Electronic Supplementary Material**

**Novel Nanostructure Approach for Antibiotic Decomposition in a Spinning Disc Photocatalytic Reactor**

Saeid Fallahizadeh^a,b^, Mahmood Reza Rahimi^c,^*, Mitra Gholami^a,b,^*, Ali Esrafili^a,b^, Mahdi Farzadkia^a,b^, Majid Kermani^a,b^

a Research Center for Environmental Health Technology, Iran University of Medical Sciences, Tehran, Iran

b Department of Environmental Health Engineering, School of Public Health, Iran University of Medical Sciences, Tehran, Iran

c Process Intensification Laboratory, Department of Chemical Engineering, Yasouj University, Yasouj, 75918-74831, Iran

*Corresponding authors. E-mail address: M.R Rahimi (mrrahimi@yu.ac.ir); M. gholami ([gholamim@iums.ac.ir](mailto:gholamim@iums.ac.ir))


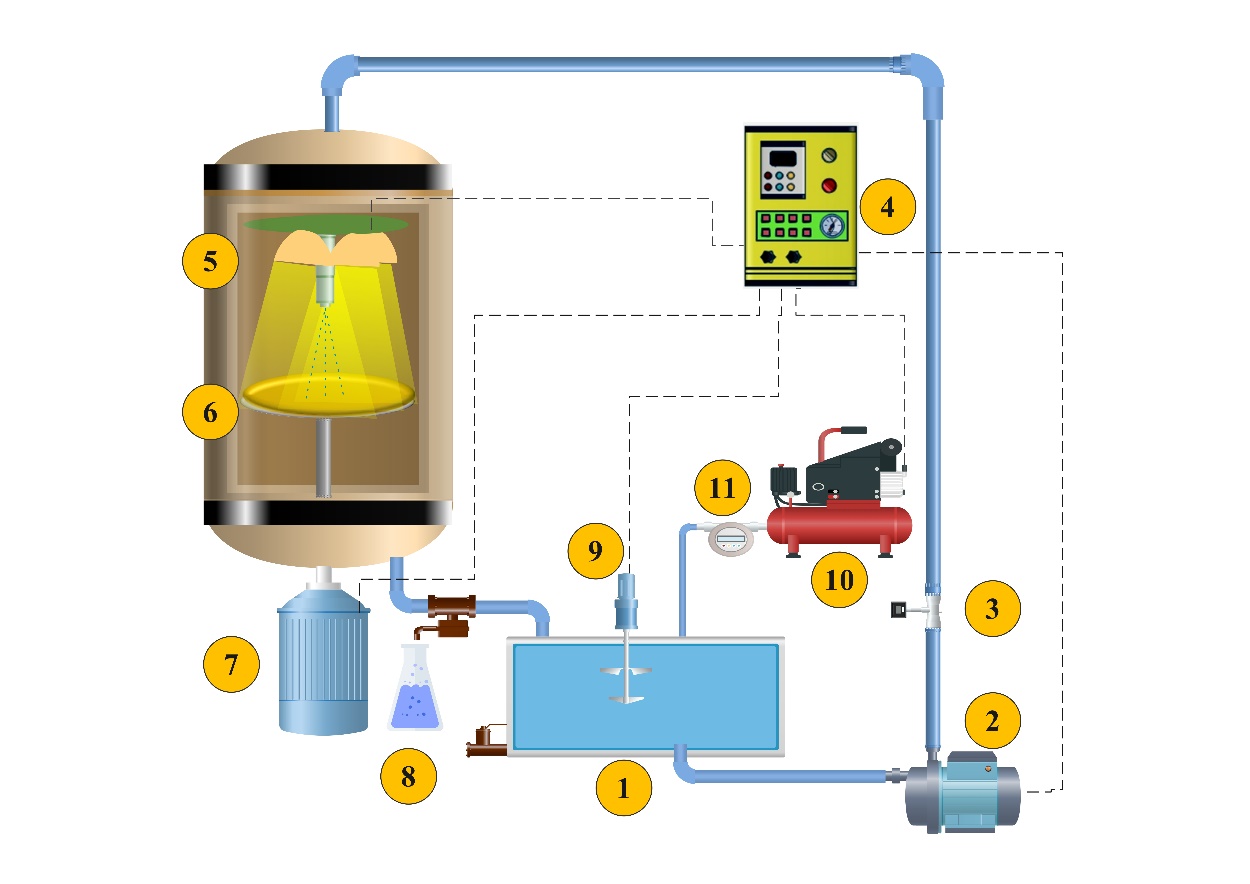


Fig. S1 Schematic details of the SDPR in this research, 1: Reservoir tank, 2: Fluid pump, 3: Flowmeter, 4: Electrical power box, 5: LED visible light source, 6: Spinning disc, 7: Motor for rotating the disc, 8: sampling location, 9: Mixer, 10: Air compressor, 11: Flowmeter.


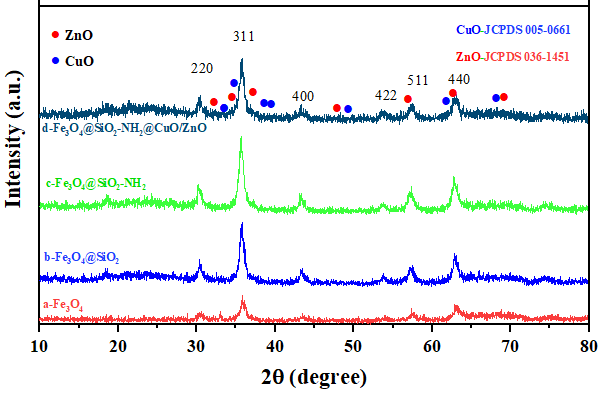


Fig. S2.The XRD patterns of Fe_3_O_4_, Fe_3_O_4_@SiO_2_ core-shell, Fe_3_O_4_@SiO_2_-NH_2_ core-shell and Fe_3_O_4_@SiO_2_-NH_2_@CuO/ZnO core-shell nanostructures


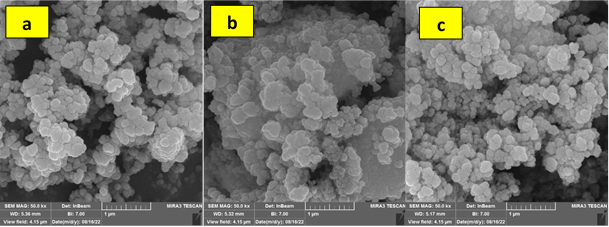


Fig. S3 FE-SEM images of (a) Fe_3_O_4_@SiO_2_, (b) Fe_3_O_4_@SiO_2_-NH_2_, and (c) Fe_3_O_4_@SiO_2_-NH_2_@CuO/ZnO Core-Shell.
